# Age-Associated Changes in the Spectral and Statistical Parameters of Surface Electromyogram of *Tibialis Anterior*

**DOI:** 10.1155/2016/7159701

**Published:** 2016-08-17

**Authors:** Ariba Siddiqi, Sridhar Poosapadi Arjunan, Dinesh Kant Kumar

**Affiliations:** Biosignals Lab, Electrical and Computer Engineering, RMIT University, Melbourne, VIC 3000, Australia

## Abstract

Age-related neuromuscular change of Tibialis Anterior (TA) is a leading cause of muscle strength decline among the elderly. This study has established the baseline for age-associated changes in sEMG of TA at different levels of voluntary contraction. We have investigated the use of Gaussianity and maximal power of the power spectral density (PSD) as suitable features to identify age-associated changes in the surface electromyogram (sEMG). Eighteen younger (20–30 years) and 18 older (60–85 years) cohorts completed two trials of isometric dorsiflexion at four different force levels between 10% and 50% of the maximal voluntary contraction. Gaussianity and maximal power of the PSD of sEMG were determined. Results show a significant increase in sEMG's maximal power of the PSD and Gaussianity with increase in force for both cohorts. It was also observed that older cohorts had higher maximal power of the PSD and lower Gaussianity. These age-related differences observed in the PSD and Gaussianity could be due to motor unit remodelling. This can be useful for noninvasive tracking of age-associated neuromuscular changes.

## 1. Introduction

Age-associated decline in muscle strength of the Tibialis Anterior (TA) is very significant and has been found to increase the risk of falls [1]. A preceding neuromuscular change to muscle strength decline in the TA is motor unit remodelling [2], which results in reduced number but increased size of motor units [3]. Investigating the changes of the muscle activity of TA noninvasively would be useful to detect signs of muscle weakness in the elderly.

Surface electromyogram (sEMG) is an easy-to-record noninvasive signal of the muscle activity. It is generated by the superposition of the electrical potential induced by the motor unit action potentials (MUAP) in the muscle. Recent studies have demonstrated age-associated changes of the sEMG [4–6]. One cause of this can be due to change in the shape of MUAP due to neuropathy [7]. However, there are a number of factors that can affect sEMG, and it is essential to identify suitable features of the signal to evaluate these changes.

Zhao and Li [8] used a sEMG model of the biceps brachii and determined the effect of the number of active motor units and firing rate schemes on the higher-order statistics of the signal: Gaussianity and linearity. It was shown that the number of active motor units is correlated with Gaussianity of the sEMG signal [8] and it was demonstrated that the decreased number of motor units due to motor unit remodelling can be studied with Gaussianity. This concept has been experimentally validated for the* m. biceps brachii*, showing increasing Gaussianity of the sEMG with increasing force [9–11].

Age-associated change in Gaussianity has been studied for the biceps brachii [9]; however, there is no reported study that has been performed for the TA and recently authors have reported the sEMG features related to age-related motor unit remodelling in TA [12]. Increased amplitude of the MUAP with a corresponding increased sEMG RMS has been observed with age in the TA, which was indirectly related to motor unit remodelling [3]. This finding has been further confirmed by Fling et al. [13] who found increased macro EMG amplitude in older cohorts indicative of increased motor unit size. Nonetheless, the amplitude of the sEMG is affected by the number of extrinsic [14] and intrinsic factors [14, 15] and neuromuscular evaluations based solely on the sEMG amplitude can be misleading.

This study reports the age-associated change in the Gaussianity of sEMG of the TA muscles. It has developed the baseline of the age-associated changes in the sEMG of the TA muscle by experimentally investigating the difference in sEMG features between the younger and older cohorts. The experiments were conducted for different levels of contraction measured based on the individual's maximal voluntary contraction (MVC).

## 2. Materials and Methods

### 2.1. Participants

Eighteen younger and 18 older volunteers (details in Table 1) with no clinical history of neuromuscular disease or ankle injury participated in this study. The experimental protocol was approved by RMIT University Human Research Ethics Committee and was in accordance with Helsinki Declaration (revised 2004).

### 2.2. Mechanical and sEMG Recording Procedures

**Figure 1:**
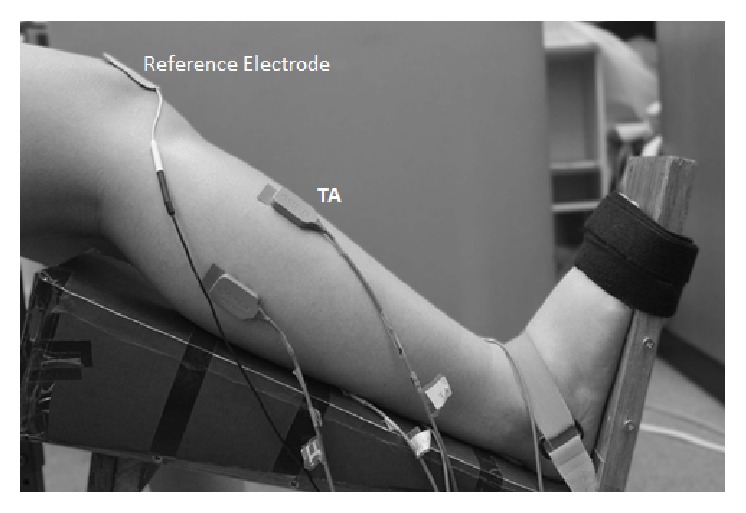
Experimental setup for isometric dorsiflexion of the ankle and the sEMG electrode placement for Tibialis Anterior.

Participants were seated in a sturdy chair with the right leg strapped to a support such that the hip, knee, and ankle were fixed at 90°, 140°, and 90° (neutral position), respectively. The angles were measured using generic goniometer. A force sensor, SM-100 type (Interface, Arizona, USA), was attached to measure the isometric force applied to the fixed footplate. The left leg was planted firmly on the ground. To ensure absence of foot or toe movement during dorsiflexion, the foot and ankle were secured with straps to the footplate [16] (Figure 1).

Myomonitor 4 (Delsys, Boston, USA) was used to record the EMG activity which had a gain of 1000, CMRR of 92 dB, and bandwidth of 20–450 Hz, with 12 dB/octave roll-off. The sampling frequency was set to 1000 Hz with resolution of 16 bits/sample. The Delsys single-channel active differential silver bar (1 mm × 1 mm) surface electrodes with an embedded preamplifier and interelectrode distance of 10 mm were used. The skin at the electrode locations was shaved, abraded, and cleansed with an alcohol swipe. EMG and force recordings were performed concurrently during the experiment.

### 2.3. Electrode Placements

The electrode placement and skin preparation were done in accordance with SENIAM recommendations [17]. EMG activity was recorded from the dorsiflexor, TA. The surface electrode was placed at 1/3rd on the line between the tip of the fibula and the tip of the medial malleolus [17] and the ground electrode was placed at the patella (Figure 1).

### 2.4. Experimental Protocol

Prior to maximal voluntary contraction (MVC) measurement for isometric dorsiflexion (DF), participants were trained to elicit their true MVC with visual force feedback on the screen. After significant rest and self-administered massage, they repeated this twice and recordings were accepted if the difference was less than 5% from each other. Minimum two-minute rest period during which the subjects massaged the muscle was given between each trial. Subsequently, they were instructed to perform two isometric DF repetitions, each of 10, 20, 30, and 50% MVC, each for 5 seconds with a two-minute rest period between each trial, with the order being randomly generated.

### 2.5. EMG Data Analysis

The following EMG features were computed to identify the age-related changes in the Tibialis Anterior muscle:

- Gaussianity: bispectral analysis.
- Power spectral density.

*(a) Gaussianity Measure*. Reduction in the number of action potentials due to reduced number of active motor units will make the sEMG signal less Gaussian [18]. The Gaussianity of a signal can be assessed by several methods, for example, negentropy [11], probability density function [19], and recently developed third-order cumulant based features [20, 21]. In this study, the Gaussianity test developed by Hinich [22] which tests the nonskewness of a time series has been used. This is because a relationship between number of active motor units and these features has been established [8].

The algorithm developed by Hinich [22] tests whether bispectrum (1) which is the Fourier transform of third-order cumulant (2) is zero. This occurs if third-order cumulant of a process is zero, leading bicoherence (3) (normalised bispectrum) to also be zero:

(1) Bω1,ω2=∑m=−∞m=∞∑n=−∞n=∞Rm,ne−jω1m+ω2n

(2) Rm,n=EXkXk+mXk+n

(3) Bnω1,ω2=Bω1,ω2Pω1Pω2Pω1+ω2,

where *P*(*ω*) is the power spectrum.

If the bispectrum, *B*(*ω*_1_, *ω*_2_), is nonzero, then the underlying process or signal is classed as a non-Gaussian process. The bicoherence, *B*_*n*_(*ω*_1_, *ω*_2_), will be a nonzero constant if the process is linear and non-Gaussian. The non-Gaussianity test statistic (Sg) is chi-squared distributed with 2P degrees of freedom when the bicoherence is zero (4). Hence, if the Sg value is determined to be consistent with a central chi-squared distribution, the null hypothesis of assumption of Gaussianity can be made:

(4) $$\begin{array}{c} \text{Sg}=\sum {\left|\;B_{n}\left(\;\omega_{1},\omega_{2}\;\right)\;\right|}^{2}. \end{array}$$

*(b) Power Spectral Analysis*. Neuromuscular changes have typically been studied using amplitude features such as root mean square (RMS) [6, 23, 24], but this suffers from amplitude cancellation [14]. The power spectrum is not influenced by amplitude cancellation [25] and can be used to study amplitude changes in the sEMG.

The power spectral density of the sEMG was calculated for epoch lengths of 512 points with a 25% overlap [26]. The maximal power (dB) of the power spectral density curve was determined.

### 2.6. Statistical Analysis

Statistical analysis was performed using MATLAB and Statistics Toolbox Release 2011a (MathWorks Inc., Massachusetts, USA). One-way ANOVA was performed on the data with age as a factor with two levels:* young* and* old*. This was repeated for each of the MVC levels: 10%, 20%, 30%, and 50%. Prior to ANOVA calculations, normality of the test data was performed using Shapiro-Wilk test at *α* = 0.05. If the test data was determined to be not normal, this was transformed using the Aligned Rank Tool (ARTool) developed by Wobbrock et al. [27] to address the need for performing factorial analysis on nonparametric data and has been verified to be accurate. All the statistical results are tabulated in Table 2.

## 3. Results

**Figure 2:**
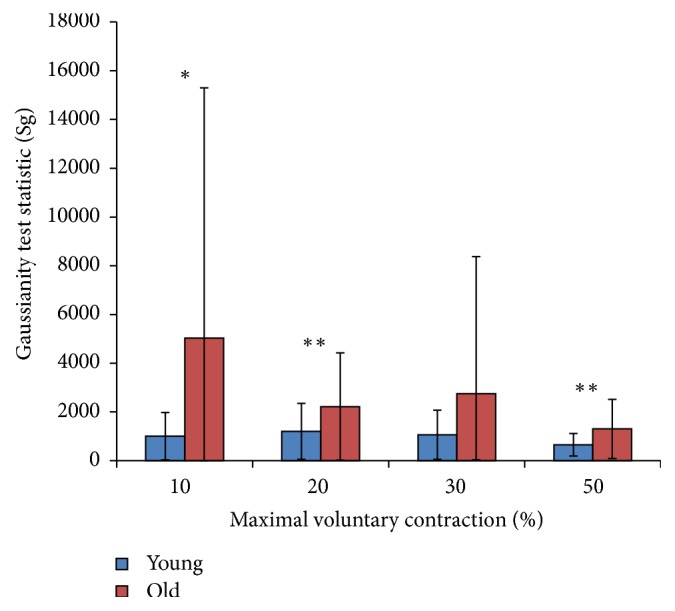
Changes in the Gaussianity test statistic value of the young and old cohort's EMG with increasing force level measured as a percentage of maximal voluntary contraction. Significant age-associated difference indicated by ^*∗*^ *p* < 0.1; ^*∗∗*^ *p* < 0.05.

**Figure 3:**
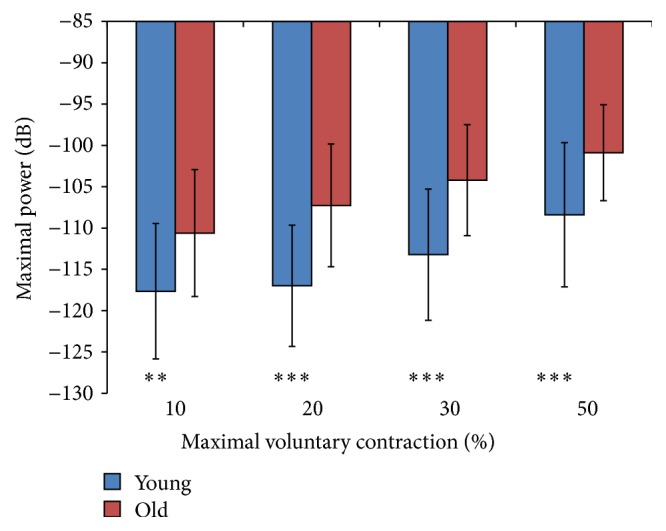
Maximal power (dB) of the EMG signal PSD as a function of force (% MVC) in young and old cohorts. Significant age-associated difference indicated by ^*∗∗*^ *p* < 0.05; ^*∗∗∗*^ *p* < 0.01.

The relationship of Gaussianity and maximal power of PSD for different levels of MVC and for the two age groups is shown in Figures 2 and 3, while the statistical description is in Table 2. These are described below.

### 3.1. Gaussianity Measure

Figure 2 illustrates the changes in the mean ± SD of Gaussianity test statistic (Sg) of sEMG recorded from the young and older cohorts with increasing force level. The results show a decrease in Sg with increasing force level, indicating that sEMG is increasingly Gaussian at higher force levels. While the change in Gaussianity was more evident for the older cohort, the younger cohort had overall higher Gaussianity at all force levels. The difference between the two age cohorts was significant (Table 2). All the Sg values from both cohorts were determined to be not normally distributed (*p* < 0.05). Therefore, the Sg values were transformed using the ARTool prior to ANOVA calculations [27].

### 3.2. Power Spectral Density (PSD)

Figure 3 shows the mean ± SD of maximal power measured (in dB) from the participant's sEMG power spectral density (PSD) for different force levels. A decibel (dB) is a logarithmic unit that can be used to represent power in a signal. As the power in the signal increases, its value in dB will become less negative. Both cohorts display increasing maximal power of their sEMG with force, but the older cohorts always maintained higher maximal power than the young. The maximal power of the sEMG's PSD was determined to be normally distributed for both cohorts, with the exception of the young cohorts' value at 50% MVC. Both cohorts' maximal power values at 50% MVC were transformed using the ARTool [27] prior to ANOVA calculations. A main effect for age was found at 10% MVC (*p* < 0.05), 20% MVC (*p* < 0.001), and 50% MVC (*p* < 0.001) (Table 2).

## 4. Discussion

This paper has established the baseline for the age-associated changes to the sEMG of the TA muscle. This muscle was investigated because earlier studies have identified changes in this muscle to be very significant [28–30] and it is a commonly used muscle model for dystrophy research [31] and the older cohorts have lower mean motor unit firing rates for TA when compared to young adults [32]. It has experimentally investigated the age-related change in sEMG of TA muscle using two features: Gaussianity and maximal power of PSD. From the results, it is confirmed that the maximal power of the PSD and Gaussianity increase with increased levels of muscle contraction. Significant differences in the PSD and Gaussianity of the sEMG recorded from the TA muscle between the younger and older cohorts were also observed which shows that age-related changes can be assessed with these features. To interpret the results of these experiments, these features need to be examined individually.

Maximal power of the PSD is an indicator of increased size or firing rate of the motor unit. From the literature, it is evident that firing rate reduces with age [2, 33]; thus, the age-associated increase in maximal power of the PSD can only be attributed to the size of the motor units. Based on the central limit theorem, a system distribution becomes closer to Gaussian as the number of independent components increases [34]. Decreases in the Gaussianity test statistics (Sg) value show the system to be closer to Gaussian distribution and indicate a larger number of motor units. Reduction of Sg of sEMG can be interpreted as reduced number of motor units.

Increasing force demands are met through two schemes: recruitment and rate coding. Both of these will increase the MUAP content of the sEMG which will increase the PSD amplitude [6, 9] and Gaussianity of the EMG signal [9–11]. This is readily observed in this study. This is also supported by Istenič et al.'s [18] study who suggested that increased force would lead to greater number of MUAP contributing to the amplitude distribution and hence higher Gaussianity.

Conversely, decrease in the number of active motor units would contribute a lesser number of MUAP to the EMG signal and lead to a narrower amplitude distribution centered about zero [18]. Since Gaussianity is correlated with the number of active motor units, its decrease in the older cohorts' sEMG is indicative of decreased number of motor units in the TA.

Likewise, the older cohorts' sEMG had higher maximal power of the PSD, in agreement with other studies on sEMG amplitude [2, 13, 35]. Motor unit remodelling is the increased MUAP amplitude due to increased innervation ratio [3, 36] and could explain the increase in sEMG amplitude [2, 13, 35].

## 5. Conclusion

This study has investigated the effect of age on the Gaussianity and the PSD amplitude of the sEMG for the Tibialis Anterior at different force levels. SEMG of the older cohorts had higher amplitude and more non-Gaussianity, which was especially evident at submaximal contractions. The combination of these features may be associated with motor unit remodelling and would be useful for noninvasively tracking neuromuscular changes with age and implementing rehabilitation strategies to prevent strength degradation. While this study has developed the baseline for age-associated changes of TA muscles, there is a need for identifying the difference between the active elderly and those at risk of falls, or the “easy fallers.” There is also a need for establishing the relative contribution of other lower limb muscles in the decline in strength during standing or walking.

## Figures and Tables

**Table 1:** Details of the participants: number, age, height, and body mass index.

Group	Age (years)	Height (cm)	Body mass index
Young (*n* = 18)	26.1 ± 2.9 (20–30)	166.7 ± 8.9	22.3 ± 2.9
Old (*n* = 18)	67.7 ± 8.1 (60–85)	163.2 ± 9.1	26.0 ± 3.9

**Table 2:** ANOVA significant statistical results for the EMG features.

EMG feature	Effect	MVC (%)	*F*(1,35)	*p* value
Gaussianity test statistics (Sg)	Age	10	3.35	0.0760
		20	5.18	0.0292^*∗*^
		50	4.84	0.0347^*∗*^

Maximal power (dB)	Age	10	7.07	0.0119^*∗*^
		20	15.58	0.0004^*∗∗*^
		30	13.5	0.0008^*∗∗*^
		50	9.96	0.0033^*∗∗*^

Significant difference between young and old cohorts indicated by ^*∗*^ *p* < 0.05 and ^*∗∗*^ *p* < 0.01.
